# Composite Behavior of RC-HPFRC Tension Members under Service Loads

**DOI:** 10.3390/ma14010047

**Published:** 2020-12-24

**Authors:** Carlos Zanuy, Pedro Javier Irache, Alejandro García-Sainz

**Affiliations:** Department Continuum Mechanics and Structures, Universidad Politécnica de Madrid, ETS Ingenieros de Caminos, 28040 Madrid, Spain; pj.irache@alumnos.upm.es (P.J.I.); alejandro.garcia.sainz@alumnos.upm.es (A.G.-S.)

**Keywords:** composite members, HPFRC, tension, strengthening, cracking

## Abstract

A significant increase of the use of high-performance fiber-reinforced concrete (HPFRC) to strengthen reinforced concrete structures (RC) has been noted for the past few years, thereby achieving composite RC-HPFRC elements. Such a technique tries to take advantage of the superior material properties of HPFRC in the ultimate and service load regimes. Many of the existing works on RC-HPFRC elements have focused on the strength increase at the ultimate load state and much less effort has been devoted to the serviceability response. The in-service performance of RC structures is governed by the behavior of the tension chord, which determines the crack pattern (crack widths are critical for durability) and deformations. The presence of HPFRC is supposed to improve serviceability due to its strain-hardening and tension-softening capacities. In this paper, the experimental analysis of composite RC-HPFRC tension members is dealt with. Specimens consisting of a RC tie strengthened with two 35 mm thick HPFRC layers have been subjected to loads in the service range so that the deformational and cracking response can be analyzed. The HPFRC has been a cement-based mortar with 3% volumetric amount of short straight steel fibers with a compressive and tensile strength of 144 MPa and 8.5 MPa, respectively. The experiments have shown that RC-HPFRC has higher stiffness, first cracking strength and reduced crack widths and deformations compared to companion unstrengthened RC. To understand the observed behavioral stages, the experimental results are compared with an analytical tension chord model, which is a simplified version of a previous general model by the authors consisting of 4 key points. In addition, the influence of time-dependent shrinkage has been included in the presented approach.

## 1. Introduction

**Figure 1:**
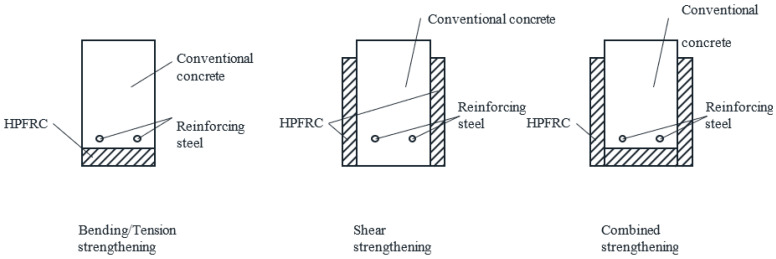
Possible schemes for strengthening a reinforced concrete cross-section with high-performance fiber-reinforced concrete (HPFRC) layers, abstracted from [4,5,6,7,8,9].

In the last years, the use of high-performance fiber-reinforced concrete (HPFRC) as strengthening material for existing concrete structures has increased significantly [1,2,3]. The most common strengthening technique has been based on the application of thin HPFRC layers on the old concrete surface, either longitudinally [4,5,6,7] or transversely [8,9,10], to improve the tension/bending or shear capacity of structural members, respectively (Figure 1). In order to ensure that the superior properties of HPFRC in tension and compression contribute to the global capacity of strengthened members, an adequate bond between the HPFRC and the old concrete is required, which can be achieved by surface treatments on the old concrete layer before the application of the fresh HPFRC or by adhesive bonding of precast HPFRC strips on the old concrete [11,12,13]. If good bond is achieved, composite action between the strengthening and existing layers can be considered.

**Figure 2:**
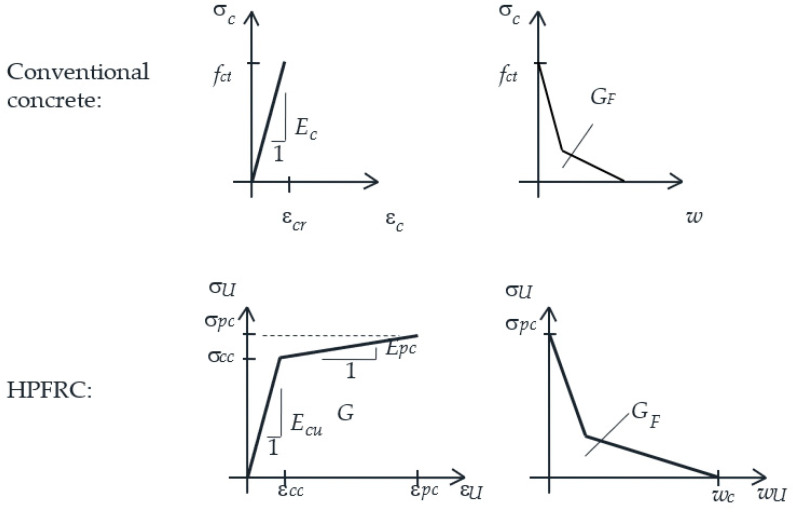
Tension behavior of conventional concrete and HPFRC, abstracted from [14,15,16,17].

The composite action of reinforced concrete (RC) members strengthened with HPFRC layers (referred to as RC-HPFRC elements hereafter) is a complex mechanism due to the different mechanical properties of both layers and their interaction. In tension, conventional concrete has a low tensile strength and negligible softening capacity, while HPFRC typically has a three-stage tensile response [14,15,16,17] (see Figure 2): (1) elastic, (2) pre-peak hardening (characterized by multiple microcracking), and (3) post-peak softening (after macrocrack localization). Therefore, perfect monolithic response of RC-HPFRC in tension is not possible as each layer enters a different behavioral stage and eventually cracks. It is necessary to analyze how the different material stages affect the global response.

Most existing works on RC-HPFRC have focused on the contribution of the HPFRC layer to the ultimate capacity of composite members, which is not only due to the utilization of the tensile capacity of HPFRC for bending-critical elements, but also includes strength gains of up to 2–3 times the resistance of unstrengthened shear-critical members due to the additional mechanisms mobilized by the RC-HPFRC interaction after interlayer detachment [18,19,20]. In contrast, much less attention has been paid to the serviceability. The serviceability of concrete structures requires crack control and limitation of deformations, which are governed by the interaction of constituent materials and global tension stiffening effect within the tension chord [21]. It is expected that strengthening with HPFRC can significantly improve the serviceability as crack widths and deformations can be reduced, especially with the use of HPFRC with strain-hardening capacity (refer to Figure 2). For conventional RC or even reinforced FRC, the tensile behavior can be described by well-known tension chord models [22,23,24], which include the stress transfer mechanism between the concrete and steel by bond stresses, thereby explaining the tension stiffening effect. Analogously, a tension chord model is necessary for RC-HPFRC elements in order to understand the mechanical contribution of the three materials involved (HPFRC, concrete and steel) and the stress transfer between them. Zanuy and Ulzurrun [25] have proposed a formulation for the tension chord of RC-HPFRC beams with tensile strengthening, but so far, no specific tests have been reported to understand the composite response of RC-HPFRC tension members. Recently, some authors tested reinforced HPFRC tension members, showing that the high tensile strength and strain-hardening capacity of HPFRC lead to improved stiffness, first cracking strength and reduced crack widths than comparative RC members with reinforcing ratios of 0.8–2.5% [26,27,28].

The present paper represents a step forward in the authors’ research towards a better understanding of the composite behavior of RC-HPFRC members. Previous research [6,25] has focused on the behavior of beam elements under bending or combined shear-bending forces. In Ref. [25], a necessity to obtain specific experimental evidence on composite tension members was put in evidence in order to complement tension chord models. Such necessity is addressed herein with a specific experimental campaign and a proposal of a simplified tension chord model for the service load domain.

The main objective of the present paper is to evaluate the benefits on stiffness, cracking strength, and ability to reduce crack widths and deformations by strengthening conventional RC with HPFRC layers. Specific tests have been carried out on composite RC-HPFRC tension members under service load levels, as well as on a companion conventional RC specimen. Crack widths and deformations are analyzed to understand the improved serviceability and the potential durability of RC-HPFRC with respect to RC with the help of a novel simplified theoretical approach able to define the behavioral phases of RC-HPFRC tension members with 4 key points. A significant novelty of the model with respect to previous approaches [25] is the introduction of the relevant role played by time-dependent shrinkage, so that the experimental results can be consistently analyzed. The effect of shrinkage is a complex phenomenon as composite members are affected by two materials (concrete and HPFRC) shrinking from different times. Herein, shrinkage effects are addressed by means of an analytical offset of the load-strain diagram and a modification of the cracking forces.

## 2. Materials and Methods

### 2.1. Material Characterization

The experimental research has studied composite RC-HPFRC tension members. The RC part has consisted of a conventional concrete prism centrically reinforced with a 16 mm ribbed steel bar (refer to Section 2.2 for details). The steel bars were B500SD quality, with characteristic yield and ultimate strength of 500 and 575 MPa, respectively, according to the Spanish code for concrete structures [29], national annex consistent with Eurocode 2 [21]. The conventional concrete was a ready mix from local supplier containing 350 kg/m^3^ of cement CEM I 42.5R, a maximum aggregate size of 12 mm and water/cement ratio of 0.5. The average compressive strength was 33 MPa at 28 days and 37 MPa at testing age (from 3 cylinders of 150 mm × 300 mm). The indirect tensile strength at testing age was 2.7 MPa (from 3 cylinders of 150 mm × 300 mm).

The HPFRC has been manufactured from a Compact Reinforced Concrete (CRC) dry-mortar premix kindly supplied by Hi-Con A/S (Hjallerup, Denmark). The mortar is a portland cement/micro silica-based binder including super plasticizer and sand aggregates of 4 mm maximum size. The mortar was mixed with the water with a water/premix weight ratio of 0.08 and a 3% volumetric amount of steel fibers in a vertical pan mixer following the instructions of the supplier. The fibers were straight, with a length of 12.5 mm, diameter of 0.3 mm, and yield strength of 2950 MPa. The average compressive strength on 15 cm cubes was 132 MPa at 28 days and 144 MPa at the age of testing of RC-HPFRC members (from 3 cubes at each age).

**Figure 3:**
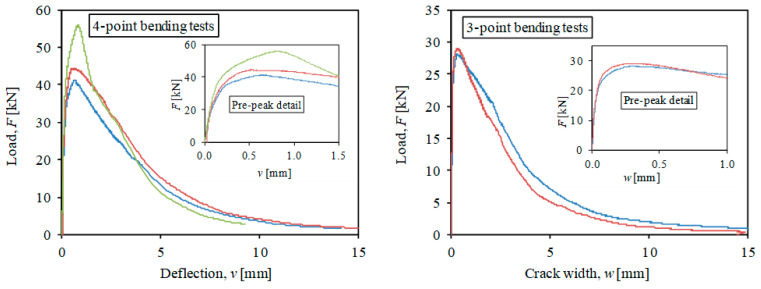
Results of flexural tests for characterization of HPFRC.

The tensile properties of HPFRC have been determined by inverse analysis [30] from 5 flexural tests on 100 mm × 100 mm × 500 mm samples at the age of testing. These flexural tests included 3 unnotched specimens tested in 4-point bending and 2 notched specimens tested under 3-point bending, in all cases with displacement control. In 4-point bending tests, the applied load and the midspan deflection are measured, while the 3-point bending tests also required measurement of the crack width evolution. Both types of tests are recommended by the protocols of [31,32] in order to capture the pre-peak hardening and the post-peak softening stages. The measurements of flexural tests are represented in Figure 3. The inverse analysis technique proposed by López et al. [30] has been used to derive the tensile properties and the results are listed in Table 1 in terms of average and coefficient of variation. As it can be noted, the HPFRC had a significant strain-hardening stage until a strain of 2.5‰. According to the material properties, some reports would classify this HPFRC as UHPFRC (ultra-high-performance fiber-reinforced concrete) [31,33].

### 2.2. Experimental Configuration

**Figure 4:**
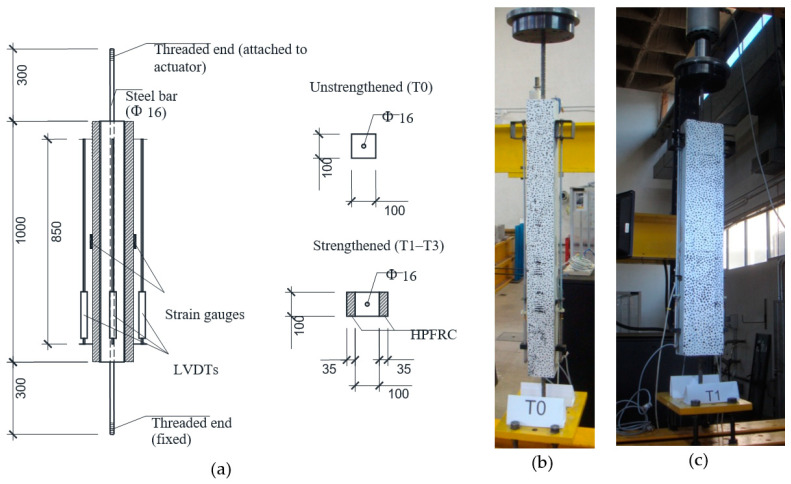
(**a**) Test set-up, instrumentation and geometry of specimens (dimensions in mm); (**b**) View of reference unstrengthened test T0 at the laboratory (side with visible speckle pattern for DIC); (**c**) Analogous view of composite test T1.

The experimental campaign included four tests on tension members. A reference test (T0) consisted of a RC specimen, while the other tests (T1, T2 and T3) were composite RC-HPFRC tension members. Therefore, test T0 can serve as a reference to be compared with the response of strengthened elements T1–T3. The dimensions of specimens and set-up configuration are represented in Figure 4. Specimen T0 has consisted of a 1600 mm long steel bar of 16 mm diameter, embedded by a conventional concrete prism of 100 mm × 100 mm × 1000 mm. The embedded length of 1000 mm allows for the formation of a sufficient number of cracks along the tension chord. The steel bar was centrically placed on the concrete prism. The free ends of the steel bar were threaded to allow their fixation to the reaction frame (lower end) and the hydraulic actuator (upper end). Specimens T1–T3 were manufactured by applying two HPFRC strengthening layers on two opposite sides (to keep the symmetry of the specimens and avoid bending effects during the tests) of RC tension members previously casted with the same configuration as specimen T0. The thickness of the HPFRC layers was 35 mm, in agreement with recommended values of the strengthening thickness to optimize the material interaction and economic use of HPFRC [5].

**Figure 5:**
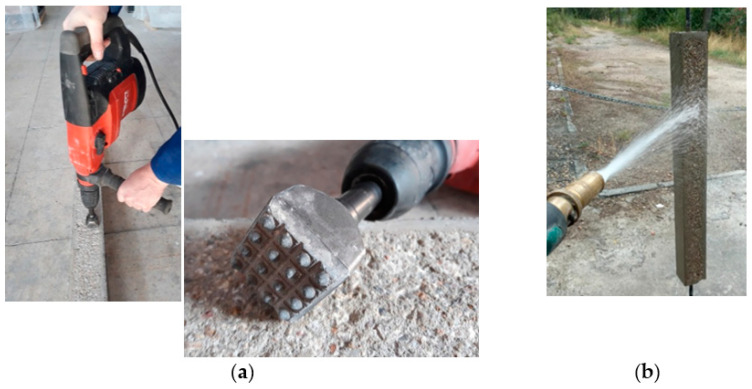
Overview of treatment on concrete surfaces: (**a**) Bush-hammering; (**b**) Surface cleaning.

The HPFRC strengthening layers were applied when the age of the RC was 60 days. The concrete surfaces were prepared by bush-hammering to achieve adequate roughness and facilitate composite action of HPFRC and conventional concrete [11]. The application of the bush hammer can be observed in Figure 5, as well as the subsequent surface cleaning to remove free or poorly attached particles. Before pouring the HPFRC on the treated concrete surfaces, they were cleaned again and kept moist.

The specimens were stored in horizontal position, with the HPFRC layers at the bottom and top sides, in laboratory environment until the testing date. The tension tests were carried out 88 days after application of HPFRC, i.e., the total age of the conventional concrete was 148 days.

The specimens were tested in tension with displacement control at a rate of 0.02 mm/s applied from the upper end with a hydraulic actuator (Servotest, Surrey, UK), until a total deformation sufficient to cover the in-service range of concrete structures (strains of 1.5–2.0‰). The transducers used for the instrumentation were the following (refer to Figure 4): 3 LVDTs to measure the elongation over a length of 850 mm (a 4th LVDT on the remaining side was not placed in order to allow for taking the pictures of a speckle pattern necessary for digital image correlation, Section 2.3), 2 strain gauges to measure the local strains at the midsection of the specimens, and a 40-ton load cell installed in the hydraulic actuator. The employed protocol for testing tension members with displacement control and measurement of strains has been validated historically by a good number of researchers, e.g., [34,35,36].

All specimens were manufactured and tested at the Laboratory of Structures of the Technical University of Madrid, Spain (UPM).

### 2.3. Digital Image Correlation

Digital image correlation (DIC) is a powerful photogrammetric technique which shows the deformation of a 2-D plain surface by taking a sequence of photographs of a speckle pattern (DIC of 3-D solids is also possible with the simultaneous use of 2 cameras). In the case of the RC-HPFRC specimens studied in this paper, one of the lateral sides with both the RC and HPFRC layers visible was painted with a speckle pattern consisting of randomly placed black points of 1–3 mm on a white background (refer to Figure 4b,c). The photographs were taken with a Nikon D90 camera (Nikon, Tokyo, Japan) equipped with lens Nikon 18–200 mm f/3.5–5.6 with a resolution of 4288 × 2848 px. The resulting px/mm equivalence is 1 px = 0.3 mm, approximately. The camera was placed perpendicularly to the analyzed surface in front of the specimens. The photographs were taken with a frequency of 0.2 fps (1 picture every 5 s). The DIC analysis has been performed with GOM Correlate software (v2018, GOM, Braunschweig, Germany) [37], with a facet size of 40 px and a point distance of 24 px. The software can detect the position of each facet, thereby providing the displacement field of the deformed surface, which allows the determination of the relevant parameters of concrete structures under service loads (strains, position of cracks, and crack widths). With the employed DIC configuration, the minimum crack width detected is around 0.03 mm.

## 3. Experimental Results

**Figure 6:**
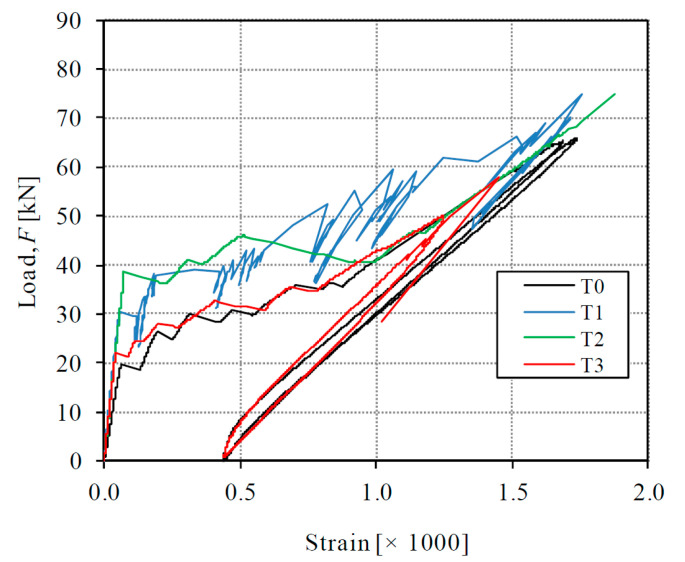
Load-strain curves of tested specimens.

The global behavior of tested specimens can be represented by the load-deformation diagrams of Figure 6. It can be noted in the graphic that an unload-reload cycle was applied in specimens T0 and T3 in order to observe the formation of permanent strains, but this aspect will not be dealt with in the present paper. The behavior of all specimens was initially linear until the first crack formation. As it can be observed, the stiffness of the initial uncracked state was significantly different from the subsequent cracked state. As will be detailed in Section 4.1, further stages can be distinguished with the help of a theoretical model. In Figure 6, the deformation is given by the average strain, which has been obtained by dividing the elongation measured with the LVDTs by the gauged length (850 mm). The first linear behavior corresponds with the elastic uncracked state of the specimens and finishes with the formation of a first transverse crack. The second experimental part has a smaller stiffness and corresponds to the crack formation and stabilized cracked behavior. According to results, the contribution of the HPFRC layers causes a stiffer behavior of the strengthened specimens T1–T3 than the reference RC specimen T0 from the beginning of the tests. For all values of the externally applied load, the average strain of specimens T1–T3 was smaller than the one of T0.

The initial stiffness of the specimens, given by the slope of the first elastic stage, was 528 MN for T1–T3 (on average) and 318 MN for T0, which means a 66% stiffness increase provided by the contribution of the HPFRC layers. In this first stage, the composite behavior was monolithic because there were no cracks. Therefore, the stiffness increase can be attributed to the elastic stiffness of the HPFRC layers (refer to Section 4.1).

**Figure 7:**
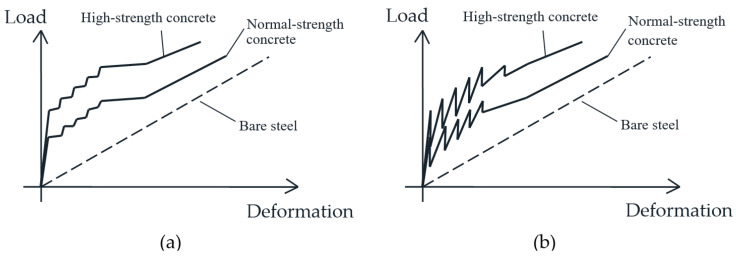
Differences in load-deformation diagrams of tension members subjected to imposed load (**a**) or imposed displacement (**b**) [38].

The average value of the first cracking load has been 30.0 kN for specimens T1–T3 and 19.3 kN for specimen T0, which means a significant 55% additional cracking strength achieved by the addition of the HPFRC layers. After the first crack formation, the global stiffness of the second stage of all specimens logically decreased. The average stiffness of the second stage, calculated as the slope of a secant line of the cracked part of the tests, was 24.2 MN for specimens T1–T3 (on average) and 27.9 MN for T0. The smaller secant stiffness of strengthened specimens is due to the fact that the second part departs from a higher cracking strength, but at the end of the service range their behavior tries to approximate the response of RC member, as the bridging capacity of the HPFRC through cracks and the tension stiffening contribution diminishes. Due to the testing procedure with displacement control, subsequent crack formation in specimens is accompanied by moderate load releases and subsequent reloading, which can be observed through some load oscillations in the diagrams of Figure 6. Such load oscillations were more notable in tests T0 and T1. Classic handbooks for structural concrete [38] have noted that such oscillations can be expected when tension members are tested under displacement-control instead of load-control, as represented in Figure 7. The analysis of local strain measurements indicates that those load oscillations have been due to formation of cracks in the conventional concrete and progressive microcracking in the HPFRC.

**Figure 8:**
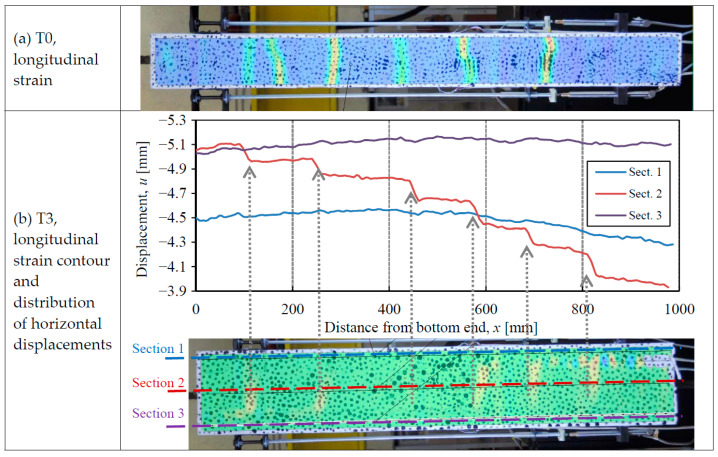
Crack detection with the help of DIC (**a**) With longitudinal strain contour (specimen T0, *F* = 40 kN); (**b**) Longitudinal strain contour supported by horizontal displacements (specimen T3, *F* = 45 kN).

The number of cracks formed in the specimens has been detected with the help of the DIC technique. Two methods can be used. On the one hand, the contours of the longitudinal strains derived from the DIC analysis can be obtained, as plotted in Figure 8a. Though the strain pattern provided by the DIC does not actually represent real strains (cracks are discrete interruptions of the continuum, whence strains have no physical sense over them), the concentration of high longitudinal strains clearly means the formation of cracks. On the other hand, the detection of cracks with strain contours can be confirmed by a study of the longitudinal displacement distribution along different axes, as plotted in Figure 8b: sections 1 and 3 along the axes of the two HPFRC layers, and Section 2 along the RC axis of the specimen. Localized displacement jumps indicate a separation of the two mouths of a single crack. Moreover, the value of the displacement jumps represents the crack width. This technique allows us to obtain the width of the cracks at the RC and HPFRC parts.

The analysis of strain contours and longitudinal displacements along sections 1 to 3 has shown the following number of cracks of each specimen: 6 (T0), 7 (T1), 8 (T2), and 6 (T3). Moreover, the average crack spacing is: 11.8 cm (T0), 12.7 cm (T1), 9.6 cm (T2) and 15.2 cm (T3). Therefore, an average value of 12.5 cm has been obtained for composite members T1–T3, which is 6% higher than the crack spacing of T0. It seems that such a slight increase has been due to the larger concrete cover of composite specimens on the strengthened sides. The crack spacing calculated with the rules of Eurocode 2 [21] would be 41.5 cm, which includes a contribution due to the concrete cover of 3.4*c* (*c* being the concrete cover). It is noted that other codes have reduced such contribution to 2*c* [29] and 1*c* [39].

It is also interesting that the strain contours and representation of longitudinal displacements (Figure 8b) have indicated that the cracks of strengthened specimens T1–T3 mainly opened in the conventional concrete, while the HPFRC layers showed an almost negligible crack width. This result shows that the width of each single crack was not the same in the concrete and the HPFRC, but it was significantly thinner in the HPFRC. Thus, the large potential of HPFRC strengthening to increase permeability and durability of concrete structures is confirmed, as the thinner cracks of HPFRC protect from the external ingress of water or corrosive agents.

**Figure 9:**
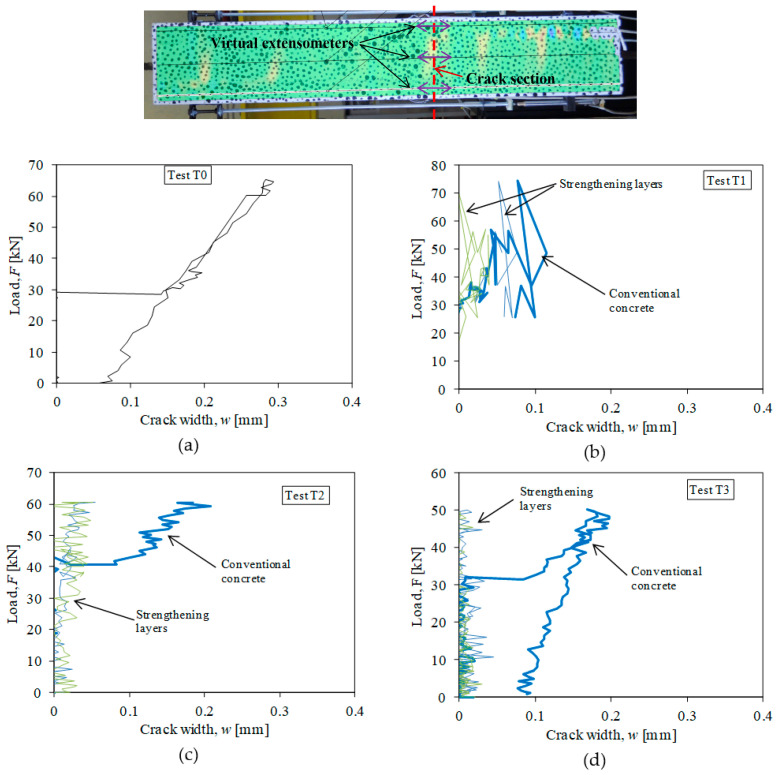
Location of virtual extensometers at a crack section and load-crack width diagrams of tested specimens: (**a**) Test T0; (**b**) Test T1; (**c**) Test T2; (**d**) Test T3.

In addition to the detection of crack positions, the DIC analysis offers the possibility to calculate the width of detected cracks and verify the benefits given by HPFRC strengthening. This can be done with the use of virtual extensometers bridging the two mouths of the cracks. Such a technique automatically provides the values of the displacement jumps sketched in Figure 8b: each virtual extensometer can determine the relative displacement between the two extensometer ends over a gauged length. It must be noted that virtual extensometers work similar to displacement transducers (LVDT) to measure crack widths [40], but they have the advantage that the crack positions do not need to be known in advance. Due to strain localization associated to concrete cracking, the position of the virtual extensometers can be fixed with the help of DIC to determine the width of localized zones where the cracks open. From the displacement fields (see Figure 8), it can be observed that the displacement jumps concentrate in lengths of 22–29 mm. Therefore, the gauged length of virtual extensometers must be larger than such lengths to capture the crack width. In the present research, each crack has been instrumented with three virtual extensometers as represented in Figure 9 (top) with a gauged length of 5 cm (i.e., 2.5 cm on each side of the cracks): one extensometer on the specimen axis to measure the crack at the RC part, and one extensometer on the axis of each HPFRC layer. The crack width is obtained as the elongation between the two ends of each virtual extensometer. Accordingly, the load-crack width diagrams of tested specimens have been obtained and the graphics corresponding to the largest (most opened) crack of each specimen are represented in Figure 9. The results confirm a double positive effect provided by the HPFRC layers. On the one hand, the width of the cracks of strengthened specimens T1–T3, measured at the conventional concrete axis, is smaller than that of the RC specimen T0: the largest crack width is of the order of 0.2 mm for specimens T1–T3, while cracks of 0.3 mm are observed in specimen T0. On the other hand, the width of the cracks of composite specimens has been very small at the HPFRC layers, of the order of 0.05 mm or smaller (microcracking). These results are very satisfactory to confirm the increased durability potential of strengthened elements.

The small crack width at the HPFRC layers is indicative of the strain-hardening capacity of the material. According to the strain hardening limit derived from material characterization (ε*_pc_* = 2.5‰ in Table 1), the HPFRC would be mostly microcracked in the serviceability domain without formation of macrocracks.

**Figure 10:**
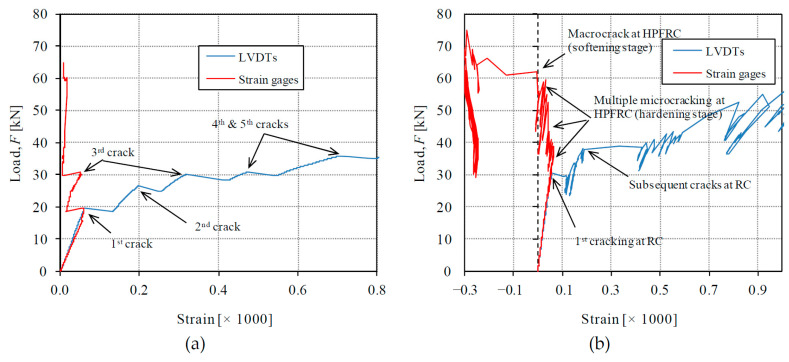
Comparison of local and global measurements with the help of strain gauges and LVDTs, respectively: (**a**) RC specimen T0; (**b**) Composite RC-HPFRC specimen T1.

The use of strain gauges (Figure 4) allows distinguishing the local behavior at a single cross-section from the global behavior detected with the LVDTs. The sequence of crack formation and the behavioral stages of conventional concrete and HPFRC can be studied by comparing local and global measurements, as represented in Figure 10. The reference unstrengthened RC response is considered first in Figure 10a; as can be noted, the formation of the first crack in the specimen produces redistribution from the concrete to the steel observed with a separation of the load-strain curves from strain gauges and LVDTs. After first crack formation, the concrete prism is divided into two parts. The formation of subsequent cracks is detected with the LVDTs, but the strain gauges are only affected by the cracks formed between the gauged cross-section and the closest previously formed crack. In the case of T0, the analysis of strain gauge measurements of Figure 10 allows us to conclude that the strain gauges have been attached at a section between the first and third cracks.

Regarding the response of composite RC-HPFRC, measurements are shown exemplarily for test T1 in Figure 10b. The signal derived from LVDTs measurements allows detecting again the subsequent crack formation, but the analysis of strain gauges (placed on the external sides of the HPFRC layers at midsection) can interestingly show the different behavioral stages of the HPFRC. First, both strain gauges and LVDTs showed the same measurement during an elastic uncracked phase, which will be referred to as Phase 1 in Section 4.1. The first cracking at the conventional concrete was detected by the separation of strain gauges and LVDTs signals at a load of 30 kN. Thereafter, local strains at HPFRC still increased linearly until a load of 36 kN (Phase 2 in Section 4.1). For the higher loads, the strain gauges showed an almost constant strain value with cyclic load releases and reloads, which was due to the formation of multiple microcracks in the HPFRC close to the gauged section during its strain-hardening stage (Phase 3 in Section 4.1). Under a load of *F* = 62 kN, the local strain decreased suddenly according to strain gauge measurements, which was indicative of the formation of a macrocrack close to the gauged cross-section and the beginning of softening stage at HPFRC.

According to the previous experimental analysis, the response of composite RC-HPFRC can be divided into clear behavioral stages. A simplified analytical model is presented in the following section in order to analyze this experimentally observed response.

## 4. Discussion

### 4.1. Theoretical Approach

In order to understand the response measured in the tests, it is necessary to compare them with a simple but consistent model. A simplified version of the composite tension chord model proposed by Zanuy and Ulzurrun [25] is presented here to characterize the different stages of RC-HPFRC elements in the serviceability range. Typically, the service range corresponds with a strain domain well before the yield limit of the steel (*f_y_*/*E_s_*). In the present paper, the analytical model is limited to the strain hardening stage of the HPFRC due to the fact that its experimentally determined upper bound (ε*_pc_* in Table 1) is here equal to the steel yield strain (2.5%). As such a strain covers the general serviceability range of tested RC-HPFRC members, the fracture process of HPFRC upon macrocrack formation including the softening stage is not dealt with in the present paper. For such deformation domains, the simplified model presented here cannot be used, but more general models (e.g., [25] or finite element simulations) are required. With the help of the general model by [25], the response of a RC-HPFRC tension member can be fully derived including the longitudinal distribution of strains and stresses between adjacent cracks.

**Figure 11:**
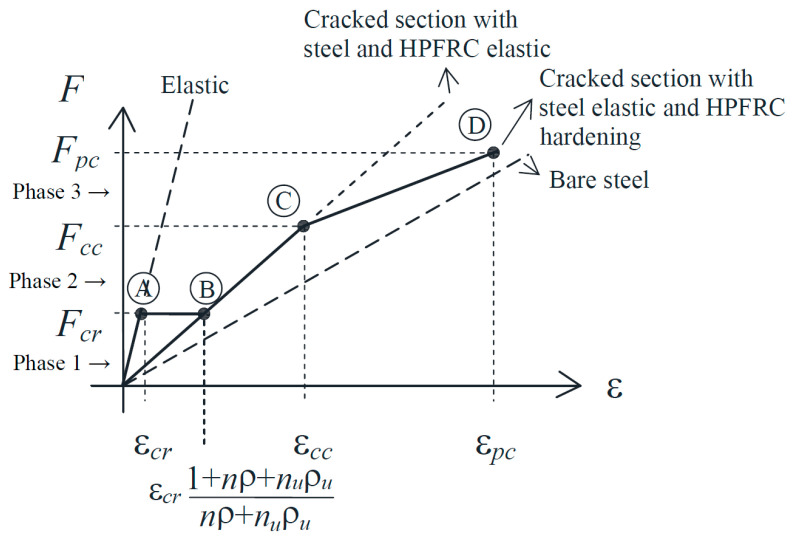
Load-strain diagram of the composite tension chord model.

The simplified version of the model presented here allows for characterizing the composite behavior by three phases and four key points (Figure 11) in terms of the load-strain diagram. The equilibrium, material, and compatibility conditions are used for the calculation of the load and strain of each key point. For the conventional concrete, it is assumed that it cannot carry tensile stresses after cracking, i.e., steel and HPFRC must carry the whole external load at cracks. The material behavior of the HPFRC is according to the one of Figure 2 and the steel is linear elastic. The model is conceptually analogous to other analytical approaches for RC members [41,42,43], but here the contribution of a third material (the HPFRC) is included in the equilibrium and compatibility at the cracked section. As the HPFRC can be either in elastic or in hardening domain, the strain compatibility is established below for each phase according to the behavioral stages of each material.

Phase 1: elastic behavior of the three materials (steel, conventional concrete, and HPFRC) until first crack formation. At this stage there is perfect bond at the interfaces (concrete-steel and HPFRC-concrete). Therefore, there are no relative slips, and the three materials have the same strain at any cross-section. Point A is characterized by the first cracking load, which takes place when the strain reaches the tensile capacity of the weakest material (conventional concrete), i.e., ε*_cr_* = *f_ct_*/*E_c_*.

(1) $$F_{A}=F_{cr}=A_{c}f_{ct}\left(1+n\rho +n_{U}\rho_{U}\right)$$

(2) $$\varepsilon_{A}=\varepsilon_{cr}=\frac{f_{ct}}{E_{c}}$$

where *n* = *E_s_*/*E_c_* and *n_U_* = *E_cU_*/*E_c_* are the transformation coefficients of steel and HPFRC with respect to conventional concrete, respectively, and *ρ* = *A_s_*/*A_c_* and *ρ_U_* = *A_U_*/*A_c_* express the relative areas of steel and HPFRC with respect to conventional concrete.

Phase 2: the concrete is cracked, while steel and HPFRC are elastic. At the cracks, the externally applied load is carried by the steel and the HPFRC. Due to concrete cracking, there is relative slip at the interfaces. For compatibility, the strain of steel and HPFRC are the same at the cracks. The first point of phase 2 (point B) corresponds to the same load as point A, but with a sudden strain increase due to the stress release at the concrete upon cracking:

(3) $$F_{B}=F_{cr}$$

(4) $$\varepsilon_{B}=\varepsilon_{cr}\frac{1+n\rho +n_{U}\rho_{U}}{n\rho +n_{U}\rho_{U}}$$

The horizontal transition between points A and B is a simplified approximation of the crack formation stage analogous to the one done in RC models [41,42,43].

The end of phase 2 takes place when the elastic limit of the HPFRC is reached (*ε_cc_*, refer to Figure 2). The corresponding load and strain of point C are as follows:

(5) $$F_{C}=F_{cc}=A_{U}\sigma_{cc}\left(1+\frac{n\rho }{n_{U}\rho_{U}}\right)$$

(6) $$\varepsilon_{C}=\varepsilon_{cc}$$

Phase 3: the concrete is cracked, the steel is elastic, and HPFRC is hardening. As in phase 2, there is relative slip at the interfaces, and the load is carried by steel and HPFRC at the cracks, where they have the same strain. The first point of phase 3 is point C. The end of phase 3 occurs when the strain-hardening limit of HPFRC (ε*_pc_*) is reached and a macrocrack opens. The load and strain of point D can be calculated by:

(7) $$F_{D}=F_{pc}=E_{s}A_{s}\varepsilon_{pc}+A_{U}\sigma_{pc}$$

(8) $$\varepsilon_{D}=\varepsilon_{pc}$$

For loads higher than *F_D_*, a numerical model or the complete general model by [25] considering the fracture process of HPFRC and the softening capacity should be used, but it is not necessary for the in-service domain studied in the present paper. Moreover, the fracture process of quasi-brittle materials is typically affected by size effect, which should be addressed properly [44]. The experiments can be affected by size effect due to strain localization in zones of a certain width. In the present paper, size effect has not been the object of study.

### 4.2. Effect of Shrinkage

**Figure 12:**
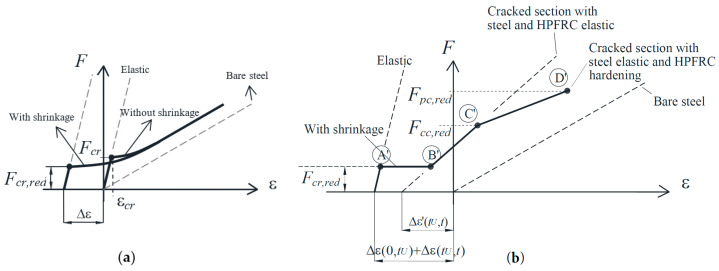
Effect of shrinkage on the behavior of tension members: (**a**) RC; (**b**) RC-HPFRC.

It has been extensively reported that the time-dependent shrinkage of concrete affects the behavior of tension members [43,45,46]. In brief, a free compressive shrinkage strain of absolute value ε*_cs_*(0,*t*) produces shortening of RC tension members and formation of self-equilibrated internal stresses (compression at the steel and tension at the concrete). Note that *t* is the time from end of concrete curing to the time of loading. According to Bischoff [47], the load-strain diagram of a RC member must be modified in two ways to consider previous shrinkage, as represented in Figure 12a: an offset of the origin and a reduction of the first cracking load with respect to the response of a member without shrinkage. The offset strain is defined in Equation (9), and the reduced cracking load is given by *F_cr,red_* in Equation (10).

(9) $$∆\varepsilon \left(0,t\right)=-\frac{\varepsilon_{cs}\left(0,t\right)}{1+n\rho }$$

(10) $$F_{cr,red}=A_{c}\left(1+n\rho \right)\left[f_{ct}\left(1-∆\sigma_{c}\right)\right],\;∆\sigma_{c}=E_{c}\varepsilon_{cs}\left(0,t\right)\frac{n\rho }{1+n\rho },\;∆\sigma_{s}=-\frac{E_{s}\varepsilon_{cs}\left(0,t\right)}{1+n\rho }$$

where Δσ*_c_* and Δσ*_s_* are the internal stresses developed in the concrete and the steel. Figure 12a shows how the shrinkage produces a modification of the uncracked elastic stage, while the bare steel curve, to which the cracked response approaches, remains unchanged. Analogous effects (initial shortening and reduction of first cracking strength) have been proposed for FRC or HPFRC tension members reinforced with steel bars and appropriate models have been formulated [28,48,49].

In case of composite RC-HPFRC members, the shrinkage effect is more complicated due to the construction sequence and the presence of two materials shrinking from different times (conventional concrete and HPFRC). Such time-dependent effects have not been considered in the previous general model [25] and it is a novelty of the present paper. The behavioral phases described in Section 4.1 (Figure 11) must be modified as follows:

*Phase 1:* Before the instant of application of the HPFRC strengthening layers (denoted as *t_U_* hereafter), the shrinkage of the conventional concrete produces the following shortening of the tension member and internal stresses:

(11) $$∆\varepsilon \left(0,t_{U}\right)=-\frac{\varepsilon_{cs}\left(0,t_{U}\right)}{1+n\rho }$$

(12) $$∆\sigma_{c}\left(0,t_{U}\right)=E_{c}\varepsilon_{cs}\left(0,t_{U}\right)\frac{n\rho }{1+n\rho },\;∆\sigma_{s}\left(0,t_{U}\right)=-\frac{E_{s}\varepsilon_{cs}\left(0,t_{U}\right)}{1+n\rho }$$

From the installation of the HPFRC layers (*t_U_*) to the time of loading (*t*), both conventional concrete and HPFRC shrink. The free shrinkage strains of conventional concrete and HPFRC are ε*_cs_*(*t_U_*,*t*) and ε*_Us_*(*t_U_*,*t*) in absolute values, respectively. The composite tension chord is then monolithically shortened by the following strain:

(13) $$∆\varepsilon \left(t_{U},t\right)=-\frac{\varepsilon_{cs}\left(t_{U},t\right)+n_{U}\rho_{U}\varepsilon_{Us}\left(t_{U},t\right)}{1+n\rho +n_{U}\rho_{U}}$$

The self-equilibrated internal stresses are developed in the three materials, as follows:

(14) $$∆\sigma_{c}\left(t_{U},t\right)=E_{c}\frac{\varepsilon_{cs}\left(t_{U},t\right)\left(n\rho +n_{U}\rho_{U}\right)-\varepsilon_{Us}\left(t_{U},t\right)n_{U}\rho_{U}}{1+n\rho +n_{U}\rho_{U}},\;∆\sigma_{s}\left(t_{U},t\right)=-E_{s}\frac{\varepsilon_{cs}\left(t_{U},t\right)+n_{U}\rho_{U}\varepsilon_{Us}\left(t_{U},t\right)}{1+n\rho +n_{U}\rho_{U}},\;∆\sigma_{U}\left(t_{U},t\right)=E_{cU}\frac{-\varepsilon_{cs}\left(t_{U},t\right)+\varepsilon_{Us}\left(t_{U},t\right)\left(1+n\rho \right)}{1+n\rho +n_{U}\rho_{U}}$$

Due to the fact that the conventional concrete already has internal stresses at *t_U_*, the creep influence has to be introduced from *t_U_* to *t*, which is done by age-adjusting its modulus of elasticity [38]:

(15) $$E_{c}\left(t_{U},t\right)=\frac{E_{c}}{1+0.85\varphi \left(t_{U},t\right)}$$

where *φ*(*t_U_*,*t*) is the creep coefficient from *t_U_* to *t*. The total modification of the elastic uncracked stage at the time of loading (*t*) can be included in the tension chord model by modifying the behavior of Phase 1 with Equations (11) and (13) for the origin offset and Equations (12) and (14) for the first cracking strength reduction (point A’ as represented in Figure 12b).

Phases 2 and 3: After the conventional concrete cracks, the load is carried by the HPFRC and the steel reinforcement. The behavior of Phases 2 and 3 must be modified by an origin offset (Δε’) and by the reduction of the load carrying capacity of the HPFRC (points C and D of Figure 11) by the internal stress (Δσ’*_U_*):

(16) $$∆\varepsilon^{\prime }\left(t,t_{U}\right)=-\frac{\varepsilon_{Us}\left(t,t_{U}\right)}{1+\frac{n\rho }{n_{U}\rho_{U}}}$$

(17) $${∆\sigma }_{U}^{\prime }\left(t,t_{U}\right)=E_{cU}\varepsilon_{Us}\left(t,t_{U}\right)\frac{n\rho }{n\rho +n_{U}\rho_{U}},{∆\sigma }_{s}^{\prime }\left(t,t_{U}\right)=-\frac{E_{s}\varepsilon_{Us}\left(t,t_{U}\right)}{1+\frac{n\rho }{n_{U}\rho_{U}}}$$

The representation of the modified points C’ and D’ of the load-strain curve is sketched in Figure 12b.

### 4.3. Analysis of Experimental Results

**Figure 13:**
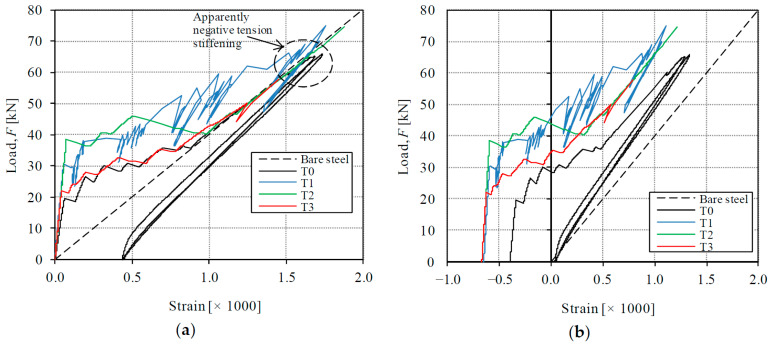
Effect of shrinkage on experimental behavior: (**a**) without shrinkage correction; (**b**) with shrinkage correction.

The experimental results are now analyzed with the theoretical formulation of Section 4.1 and Section 4.2, i.e., the effect of shrinkage is included in the study. The importance of considering the shrinkage effects is firstly demonstrated with the help of Figure 13, where the load-strain diagrams are compared with the response of the bare steel reinforcement. In Figure 13a, where the shrinkage effect is not corrected in the experimental curves, the experimental strain is larger than the one with the bare steel during the loading process, which is not possible. Such observation has been referred to as apparently negative tension stiffening effect by Zanuy [46] and is because the previous shrinkage strain has not been taken into account. A negative tension stiffening effect can only be developed during unloading stages due to the formation of residual deformations. The apparently negative tension stiffening can be solved by appropriate offsetting of the experimental curves [46,50,51]. Once the experimental curves are corrected with the shortening produced by the previous shrinkage in Figure 13b (origin offset according to Equation (9) for T0 and Equations (11) and (13) for T1–T3), the experimental strain is always smaller than the one of the bare steel bar, which is physically consistent and explains the contribution provided by concrete and HPFRC in tension between cracks. Moreover, the experimental strain of specimens T1–T3 is smaller than the one of T0 for all load levels, which shows a higher tension stiffening contribution of strengthened members due to the presence of HPFRC.

**Figure 14:**
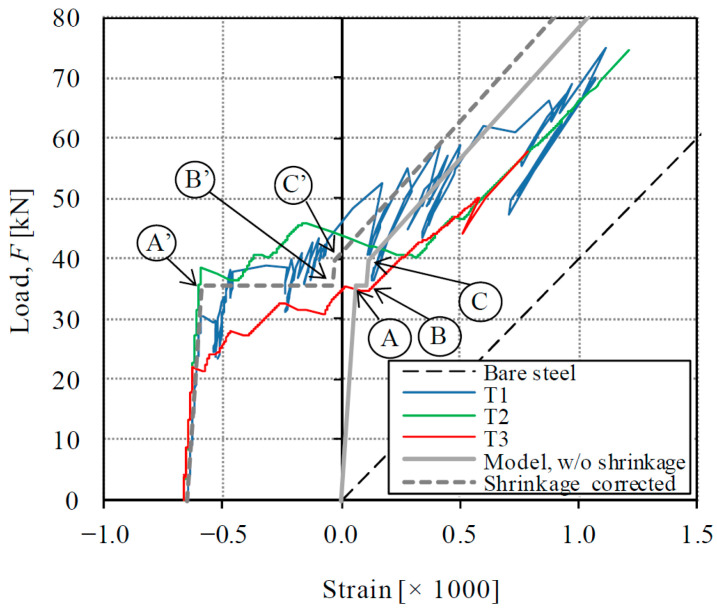
Comparison of experimental results with composite tension chord model corrected with shrinkage effect.

The comparison of experimental results of strengthened specimens T1–T3 with the model is represented in Figure 14. The key points of the model are marked in the graphic for an easy identification of the behavioral phases. As it can be noted, though the model is rather simple (it consists of 4 key points), it can provide a very good approximation of experimental results until an applied load of 60 kN. For higher loads, the experimental deformation results are larger than the theoretical one, which can be due to relative slip between the HPFRC and the conventional concrete due to the local unbonding observed at the specimen ends.

It must be noted that a factor of 0.8 has been used here for the calculation of the effective concrete area. The issue of the effective concrete area in tension has been reported as significant for members with large concrete cover where the strain uniformity across the section cannot be ensured [52]. Thus, a factor to correct the concrete area has been traditionally proposed by concrete codes [21,39]. As existing formulations have not been established for composite members, a trial-and-error has been necessary here to fit a factor of 0.8 for tested specimens.

Future research lines will focus, on the one hand, on the behavior of composite specimens in which the HPFRC strengthening layers are applied after the RC has been pre-cracked, which is a useful practical situation, and, on the other hand, on the extension of the model to ultimate limit states with the yielding of the steel reinforcement.

## 5. Comparison with Previous Beam Tests’ Results

**Figure 15:**
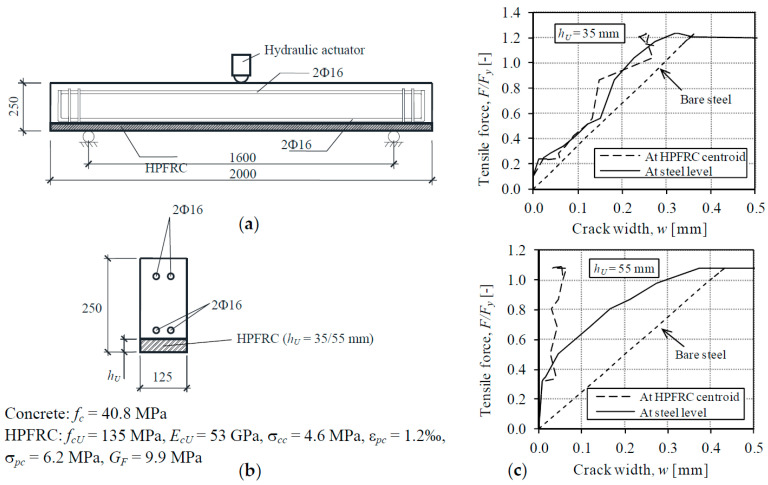
Analysis of crack evolution in previous beam tests [25]. (**a**) Dimensions in mm; (**b**) Cross-section and material properties; (**c**) Load-crack width at the tension chord of beams.

In order to further evaluate the potential benefits of the application of HPFRC strengthening layers with appreciable strain hardening capacity as those investigated in the present research, the results of previous experiments on beams tested by the authors [25] are assessed with the help of Figure 15. The beams consisted of reinforced concrete prismatic elements tested in a three-point bending configuration with a span length of 1.6 m between supports. The beams were strengthened with a HPFRC bottom layer as represented in Figure 15a. Two values of the HPFRC thickness were investigated, namely 35 and 55 mm, i.e., the later had a heavier strengthening layer with appreciable higher amount of HPFRC. The results of the tests are represented in Figure 15 by the tensile force-crack width diagrams derived as follows: on the one hand, the tensile force is the axial force acting on the tension chord at the bottom of the cross-section, consisting of the steel reinforcement, the HPFRC layer and the effective area of concrete in tension, which can be calculated as *M*/*z*, where *M* is the bending moment and *z* is the sectional lever arm The tensile force is normalized with respect to the load which produces yielding of the steel bars (*F_y_*) in order to easily focus on the serviceability range (*F*/*F_y_* < 1.0). On the other hand, the crack width has been measured at two points of the flexural crack formed at the midspan section: at the level of the longitudinal steel reinforcement and at the centroid of the HPFRC strengthening layer.

The HPFRC consisted of a CRC with 2% of steel fibers. The main difference with respect to the HPFRC used in the tension members T1–T3 was the smaller number of fibers, which was responsible for the poorer tensile properties. The small hardening capacity of the HPFRC (ε*_pc_* = 1.2‰) can be noted from the tensile properties listed in Figure 15b.

The relevance of the tensile properties can be discussed with the load-crack width diagrams plotted in Figure 15c for the two values of the HPFRC thickness. For the thickness of *h_U_* = 35 mm (the same HPFRC thickness as the one used in tension members T1–T3), the crack width at the steel reinforcement level and at the HPFRC was approximately the same from the first crack formation, without regard of the load level. Such a result is a more deficient response than the one shown in Figure 9 for specimens T1–T3, in which the crack width at the HPFRC was almost negligible due to the utilization of the strain hardening capacity of the HPFRC. The results of the bending test with HPFRC thickness of *h_U_* = 55 mm (Figure 15) shows that a thick strengthening layer can significantly reduce the crack width respect to the one with *h_U_* = 35 mm, both at the level of the longitudinal reinforcement and at the HPFRC (crack widths of 0.05 mm). Nevertheless, the increase of the consumption of high-performance material to achieve this is a significant 57% (55/35-1).

Therefore, from the comparison of the results of tension members T1–T3 with those of bending tests of [25], it can be concluded that the use of HPFRC with strain hardening capacity is an effective solution to improve the serviceability with a moderate amount of HPFRC in comparison with the application of HPFRC layers without appreciable hardening stage.

## 6. Conclusions

From the study carried out in the present paper, the following conclusions can be drawn:

- The in-service behavior of RC-HPFRC tension members is characterized by larger stiffness, higher first cracking strength and smaller crack widths than comparative unstrengthened RC members. According to the test results presented in this paper, the increase of initial stiffness and first cracking strength have been 66% and 55%, respectively. The widest crack widths measured at the axis of the specimens was 0.3 mm and 0.2 mm for RC and RC-HPFRC specimens, respectively. The experimental results showed that the tension stiffening contribution of RC-HPFRC members is significant and should not be neglected for serviceability verifications.
- The width along each single crack is smaller in the HPFRC layers than in the conventional concrete layer. According to the experimental results, crack widths were not larger than 0.05 mm at the HPFRC layers, while 0.2 mm was measured at the RC layer. Moreover, the study has demonstrated that the HPFRC can stay in the microcracking phase within the service-load range if a HPFRC mix with strain-hardening capacity is used (as in the present paper), which is rather favorable to achieve increased permeability and durability of the strengthened member.
- While in RC tension members the load is fully carried by the steel reinforcement at the cracks in the service load range, a non-negligible contribution of the HPFRC can also be expected in cracked sections of RC-HPFRC elements due to the strain hardening and softening capacities.
- From the analysis carried out in the present paper, it is fundamental to take into account the time-dependent shrinkage effect in order to fully understand the different behavioral stages of composite RC-HPFRC tension members. A simple mechanical model consisting of four key points has been proposed. An important ability of the model is the consideration of the shrinkage of the two concretes (i.e., conventional and HPFRC).
- With respect to previous research by the authors [25], this paper specifically addressed the need of experimental evidence on composite RC-HPFRC tension members. Moreover, it was demonstrated that strengthening with HPFRC with hardening capacity can significantly improve the serviceability in comparison with the results of HPFRC without appreciable hardening stage, with the use of a moderate amount of HPFRC.
- A simplified model for service load domains was presented consisting of 4 hey points, which includes the complex phenomena related with the shrinkage of the two concretes (conventional and HPFRC).

## Figures and Tables

**Table 1:** Material properties of HPFRC at testing age: average (coefficient of variation) (Refer to Figure 2 for the meaning of symbols).

*f_cU_* [MPa]	*E_cU_* [GPa]	σ_cc_ [MPa]	ε*_pc_* [–]	σ*_pc_* [MPa]	*G_f_* [MPa]
144 (0.07)	56 (0.06)	7.1 (0.18)	0.0025 (0.25)	8.5 (0.11)	14.1 (0.07)

## Data Availability

No new data were created or analyzed in this study. Data sharing is not applicable to this article.

## Glossary

*A_C_*	concrete area
*A_s_*	steel area
*A_U_*	HPFRC area
*c*	concrete cover
*E_C_*	elastic modulus of concrete
*E_CU_*	elastic modulus of HPFRC
*E_s_*	elastic modulus of steel
*F*	load
*G_F_*	fracture energy
*f_c_*	concrete compressive strength
*f_ct_*	concrete tensile strength
*f_CU_*	compressive strength of HPFRC
*f_y_*	yield strength of steel fibers
*w*	crack width
ε	strain
ε*_cs_*	free shrinkage strain of concrete
ε*_Us_*	free shrinkage strain of HPFRC
ε*_cc_*	microcracking strength of HPFRC
ε*_pc_*	hardening strain at peak strength of HPFRC
*φ*	creep coefficient
*ρ*	steel reinforcing ratio
*ρ_U_*	ratio of HPFRC area to concrete area
σ	stress
σ*_cc_*	matrix cracking strength of HPFRC
σ*_pc_*	peak strength of HPFRC
